# Correction: Rational synthesis of 10GDC electrolyte through a microwave irradiation GNP facile route for SOFC applications

**DOI:** 10.1039/d4ra90017k

**Published:** 2024-03-14

**Authors:** S. P. S. Shaikh, Chandrashekhar V. Rode

**Affiliations:** a Department of Physics, Savitribai Phule Pune University Ganeshkhind Road Pune 411007 Maharashtra India shabanamsrl@gmail.com +91 9923199822; b Chemical Engineering and Process Development Division, National Chemical Laboratory Pune 411008 India

## Abstract

Correction for ‘Rational synthesis of 10GDC electrolyte through a microwave irradiation GNP facile route for SOFC applications’ by S. P. S. Shaikh *et al.*, *RSC Adv.*, 2020, **10**, 3020–3028, https://doi.org/10.1039/C9RA09476H.

The authors regret that the contact information for the corresponding author was incorrectly shown in the original manuscript. The corrected information is as shown above.

The Royal Society of Chemistry apologises for these errors and any consequent inconvenience to authors and readers.

## Supplementary Material

